# Extended Interferon-Alpha Therapy Accelerates Telomere Length Loss in Human Peripheral Blood T Lymphocytes

**DOI:** 10.1371/journal.pone.0020922

**Published:** 2011-08-04

**Authors:** Joel M. O'Bryan, James A. Potts, Herbert L. Bonkovsky, Anuja Mathew, Alan L. Rothman, for the HALT-C Trial Group

**Affiliations:** 1 Department of Medicine, University of Massachusetts Medical School, Worcester, Massachusetts, United States of America; 2 Department of Medicine, University of Connecticut Health Center, Farmington, Connecticut, United States of America; 3 Carolinas Medical Center, Charlotte, North Carolina, United States of America; 4 Institute for Immunology and Informatics, University of Rhode Island, Providence, Rhode Island, United States of America; University of Montreal, Canada

## Abstract

**Background:**

Type I interferons have pleiotropic effects on host cells, including inhibiting telomerase in lymphocytes and antiviral activity. We tested the hypothesis that long-term interferon treatment would result in significant reduction in average telomere length in peripheral blood T lymphocytes.

**Methods/Principal Findings:**

Using a flow cytometry-based telomere length assay on peripheral blood mononuclear cell samples from the Hepatitis-C Antiviral Long-term Treatment against Cirrhosis (HALT-C) study, we measured T cell telomere lengths at screening and at months 21 and 45 in 29 Hepatitis-C virus infected subjects. These subjects had failed to achieve a sustained virologic response following 24 weeks of pegylated-interferon-alpha plus ribavirin treatment and were subsequently randomized to either a no additional therapy group or a maintenance dose pegylated-IFNα group for an additional 3.5 years. Significant telomere loss in naïve T cells occurred in the first 21 months in the interferon-alpha group. Telomere losses were similar in both groups during the final two years. Expansion of CD8^+^CD45RA^+^CD57^+^ memory T cells and an inverse correlation of alanine aminotransferase levels with naïve CD8^+^ T cell telomere loss were observed in the control group but not in the interferon-alpha group. Telomere length at screening inversely correlated with Hepatitis-C viral load and body mass index.

**Conclusions/Significance:**

Sustained interferon-alpha treatment increased telomere loss in naïve T cells, and inhibited the accumulation of T cell memory expansions. The durability of this effect and consequences for immune senescence need to be defined.

## Introduction

Telomeres are repetitive DNA sequences, consisting of hundreds to thousands of double-stranded repeats, found at both ends of every chromosome [1]. A normal cell's replicative potential has been linked to a combination of its telomere length (TL) and the ability to express telomerase. Telomerase assists in TL maintenance and slows telomere erosion during activation-induced proliferation of T lymphocytes [2], [3]. Type I interferons (IFN), in addition to anti-viral and anti-proliferative effects, inhibit expression and activity of telomerase [4]. IFN also commonly causes lymphopenia [5], which is a stimulus for homeostatic proliferation [6]. How these competing IFN responses combine to modulate TL in peripheral naïve and memory T cells is currently unclear.

Combination pegylated-IFNα (peg-IFNα) with ribavirin is the standard therapy for subjects with chronic hepatitis C virus (cHCV) infection. Unfortunately, therapy results in a sustained virologic response (SVR) in less than 50% of HCV subjects [7]. After decades of cHCV, many patients progress to liver cirrhosis and subsequent hepatic failure, and are at risk for developing hepatocellular carcinoma [8]. The Hepatitis C Antiviral Long-term Treatment against Cirrhosis (HALT-C) trial was a clinical trial designed to assess whether sustained peg-IFNα reduced the progression of liver disease in subjects who did not achieve SVR [9]. All study subjects initially received peg-IFNα plus ribavirin for 24 weeks. Subjects failing to achieve SVR were then randomly assigned either to a control, monitor-only group or to a continued peg-IFNα-treated group at a maintenance dose for an additional 3.5 years [10].

Multiple clinical measures, such as patient age, duration of infection, viral load, obesity, and liver enzyme levels have been noted to correlate to varying degrees with the IFNα treatment virologic response rate in cHCV infection [11], [12], [13], [14], [15], [16]. The role of liver hepatocyte destruction, seen as elevated blood serum levels of alanine aminotransferase (ALT), may reflect cytotoxic killing of virus infected cells by the on-going immune response. Thus serum ALT levels are used in monitoring the progression of liver damage [17]. Additionally, increasing obesity, commonly measured as body mass index (BMI), has been observed to affect telomere length in peripheral blood leukocyte subsets in otherwise healthy adults [18], [19], [20]. The availability of these clinical measures for the HALT-C cohort allowed for additional analyses in this study of their interactions with the measured peripheral blood T cell telomere lengths and telomere length changes from screening to study month 45.

In this study the primary aim was to examine the effects of long-term peg-IFNα therapy on TL in peripheral blood T lymphocytes using a flowFISH (flow cytometric fluorescence in situ hybridization) telomere length assay. Here we report significant associations between changes in TL and treatment group, patient age, serum HCV RNA level, body mass index (BMI) and alanine aminotransferase (ALT) levels.

## Results

### FlowFISH telomere length analysis in T lymphocytes

Telomere length (TL) was measured using a modified flowFISH assay [21]. We incorporated a pre-hybridization RNase treatment into the telomere flowFISH assay to minimize probe binding to telomeric RNA [22], as well as a novel set of hybridization-compatible CD4^+^ and CD8^+^ markers. This approach combined with previously described anti-CD45RA and anti-CD57 flowFISH staining allowed for quantitative estimation of average TL and frequency in T cell subpopulations. Inter-assay and intra assay variations for TL estimation were additionally assessed (see Materials and Methods).

**Figure 1:**
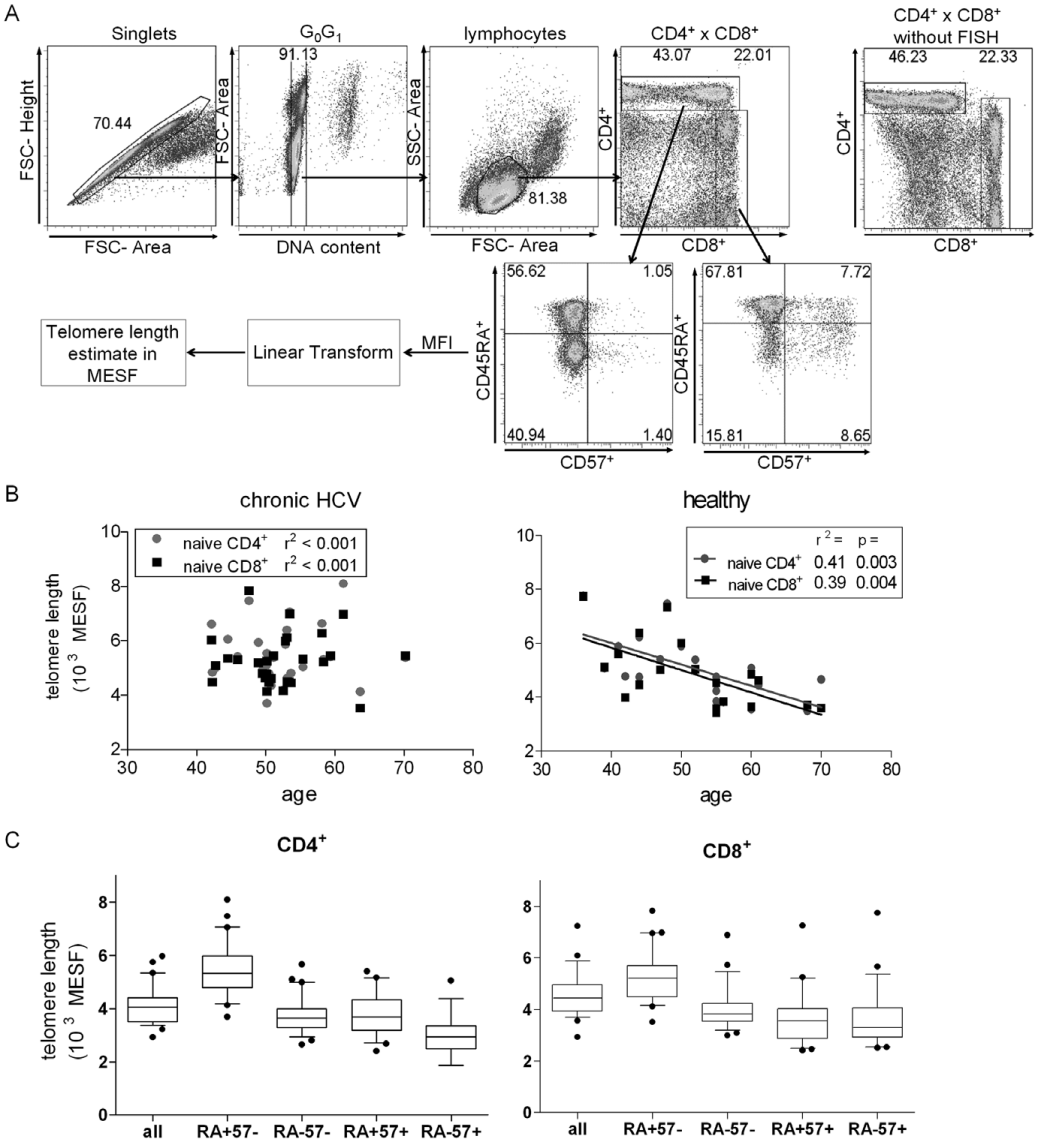
Telomere length (TL) measurement using modified flowFISH. (A) FlowFISH cytometry gating strategy used to assess TL in CD4^+^ and CD8^+^ T cells. Singlet cells in a G_0_/G_1_ (diploid DNA content) gate were further selected using a lymphocyte gate based on forward and side scatter. Flow scatter plots of CD4^+^ by CD8^+^ staining with and without flowFISH hybridization are shown. TL estimates in molecules of equivalent soluble fluorescence (MESF) in CD4^+^ and CD8^+^ populations. (B) Linear regression analysis of naïve (CD45RA^+^ CD57^−^) CD4^+^ (circles) and naïve CD8^+^ (squares) TL at screening for cHCV subjects (n = 29, left panel) and healthy, age-matched donors (n = 19, right panel) versus age at blood draw. (C) FlowFISH-determined baseline TL estimates within total CD4^+^ and CD8^+^ T lymphocytes and indicated subpopulations for all 29 HALT-C subjects at screening. Graphs are box-whisker 10–90 percentile with outliers.

Flow cytometry gating allowed discrimination of CD4^+^ and CD8^+^ T cell subsets and estimation of TL in each subset (Fig. 1A). Although the hybridization reduced the intensity of CD8^+^ staining (Figure 1A, far right panel), these two primary T cell subsets (CD4^+^CD8^−^ versus CD4^−^CD8^+^) could be discriminated. Within each subset, CD45RA discriminates memory (CD45RA^−^) T cells from the mostly naïve (CD45RA^+^) T cell subset and CD57 further enhances discrimination of naïve (CD45RA^+^ CD57^−^) T cells from CD57^+^ T cell effector-memory re-expressing CD45RA^+^ (T_EMRA_) [23].

No significant differences in clinical characteristics existed at baseline between the peg-IFNα and the no-therapy group (Table 1). Also, no differences existed in TL between these two groups at screening (Fig. S1A). However, no correlation existed between subject age and TL in the cHCV cohort (Fig. 1B left panel) in contrast to the expected inverse correlation of age and TL, an effect seen in the separate cohort of 19 healthy subjects (Fig. 1B right panel) and consistent with previous reports [24], [25].

**Table 1:** Patient characteristics at baseline and subsequent group assignment.

Patient Groupstatistic	All	IFNα therapy	No therapy	p value*
*n*	29	14	15	
Age	52.2±6.3	52.1±5.9	52.4±7.0	0.88
Gender (male:female)	24∶5	11∶3	13∶2	0.65
BMI (kg/m^2^)	30.2±5.0	32.0±4.5	28.5±5.1	0.09
Dur. infection (years)†	32.8±8.3	30.0±6.7	35.1±9.0	0.17
ALT (U/L)	86.3±42.8	95.9±47.0	77.4±37.9	0.30
log_10_HCV (copies/mL)	6.6±0.46	6.7±0.5	6.4±0.4	0.09
Ishak fibrosis score	3.5±0.95	3.6±0.9	3.4±1.1	0.48

Values are mean ± standard deviation except gender. ALT, blood alanine aminotransferase; BMI, body mass index.

*P-value for all IFNα therapy to no-therapy group comparisons by Mann-Whitney test, except gender analyzed by Fisher's exact test.

†Duration of infection unavailable for three patients, two in IFNα-therapy and one in no therapy.

Baseline TL in T cell subsets were consistent with prior reports [21], [24]; naïve (CD45RA^+^ CD57^−^) T cell TLs were greater than memory (CD45RA^−^ CD57^−^) T cell TLs, and average TL in CD57^+^ subsets were shorter than their corresponding CD57^−^ subsets (Fig. 1C). The percentage of CD57^+^ cells was higher in the CD8^+^ T cell compartment than in the CD4^+^ T cell compartment (Fig. S1B), consistent with published results [23].

### HCV viral load, body mass index correlated with TL at screening

**Figure 2:**
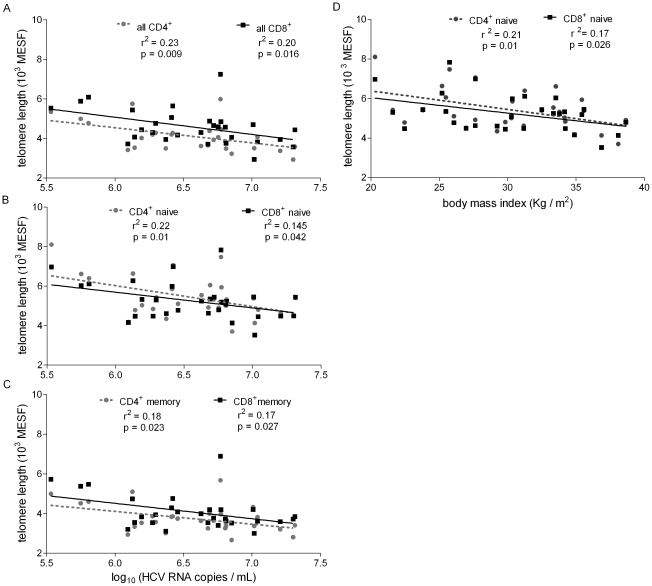
Screening T cell telomere lengths inversely correlated with hepatitis C viral RNA levels and BMI. (A) TL in total CD4^+^ (gray circles) and total CD8^+^ (black squares) T cells from the screening (S00) time point versus screening HCV RNA levels, (B) TL in naïve CD4^+^ (circles) and CD8^+^ (squares) CD45RA^+^ CD57^−^ subsets versus screening HCV RNA levels, and (C) TL in memory CD4^+^ (circles) and CD8^+^ (squares) CD45RA^−^ CD57^−^ subset versus screening HCV RNA levels. (D) Baseline body-mass index (BMI, in kilograms per meter squared) from screening assessment inversely correlated with naïve phenotype CD4^+^ (gray circles) and CD8^+^ (filled squares) T cells. Correlation (r-squared) and p values are from linear regression testing with best-fit lines as shown.

HCV viremia levels inversely correlated with TL in total CD4^+^ and CD8^+^ T cells (Fig. 2A), naïve T cells (Fig. 2B), and memory T cells (Fig. 2C) at enrollment. BMI at enrollment also inversely correlated with TL in naïve CD4^+^ and CD8^+^ T cells (Fig. 2D). However, there was no correlation between HCV viremia levels and BMI (data not shown), suggesting that BMI and viremia are independent factors associating with T cell TL in chronic HCV infection.

### Sustained IFNα therapy increased loss of telomere length

**Figure 3:**
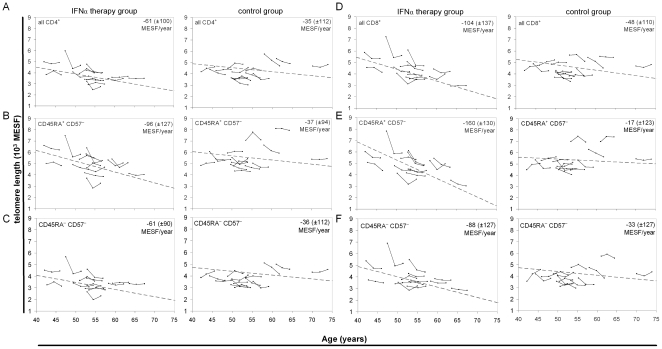
Accelerated telomere length (TL) loss in naïve T cell subsets for the IFN group. Individual TL trajectories for (A) all CD4^+^, (B) naïve CD4^+^ (CD45RA^+^ CD57^−^), (C) memory CD4^+^ (CD45RA^−^ CD57^−^), (D) all CD8^+^, (E) naïve CD8^+^ (CD45RA^+^ CD57^−^), and (F) memory CD8^+^ (CD45RA^−^ CD57^−^) T cell subsets are shown for peg-IFNα subjects (left panels) and the no-therapy, control subjects (right panels). Solid lines connect the TL of each subject at three time points plotted by age at blood draw. The dashed line on each plot derives from a linear regression based on the averaged slope and y-intercept from each individual's linear regression equation, with the average slope (± standard. deviation) in MESF per year of age shown in the upper right corner.

To determine whether IFN therapy affected telomere erosion, the rate of change in TL in CD4^+^ and CD8^+^ T lymphocyte subsets between screening (S00), month 21 (M21) and month 45 (M45) was determined for both groups (Fig. 3). The average linear regression equation-derived slopes (delta TL/year in units of MESF) showed declining TL for all subsets. The average linear regression slopes between S00 and M45 show higher average TL loss rates in the peg-IFNα treatment group across total CD4^+^ (Fig. 3A) and total CD8^+^ T cells (Fig. 3D), the naïve subsets (Figs. 3B, E) and memory subsets (Figs. 3C, F), although this difference was statistically significant only for naïve CD8^+^ T cells (p = 0.005). CD57^+^ T cells in both CD4^+^ CD45RA^+/−^ and CD8^+^ CD45RA^+/−^ subsets showed no difference in TL between treatment groups (data not shown).

**Figure 4:**
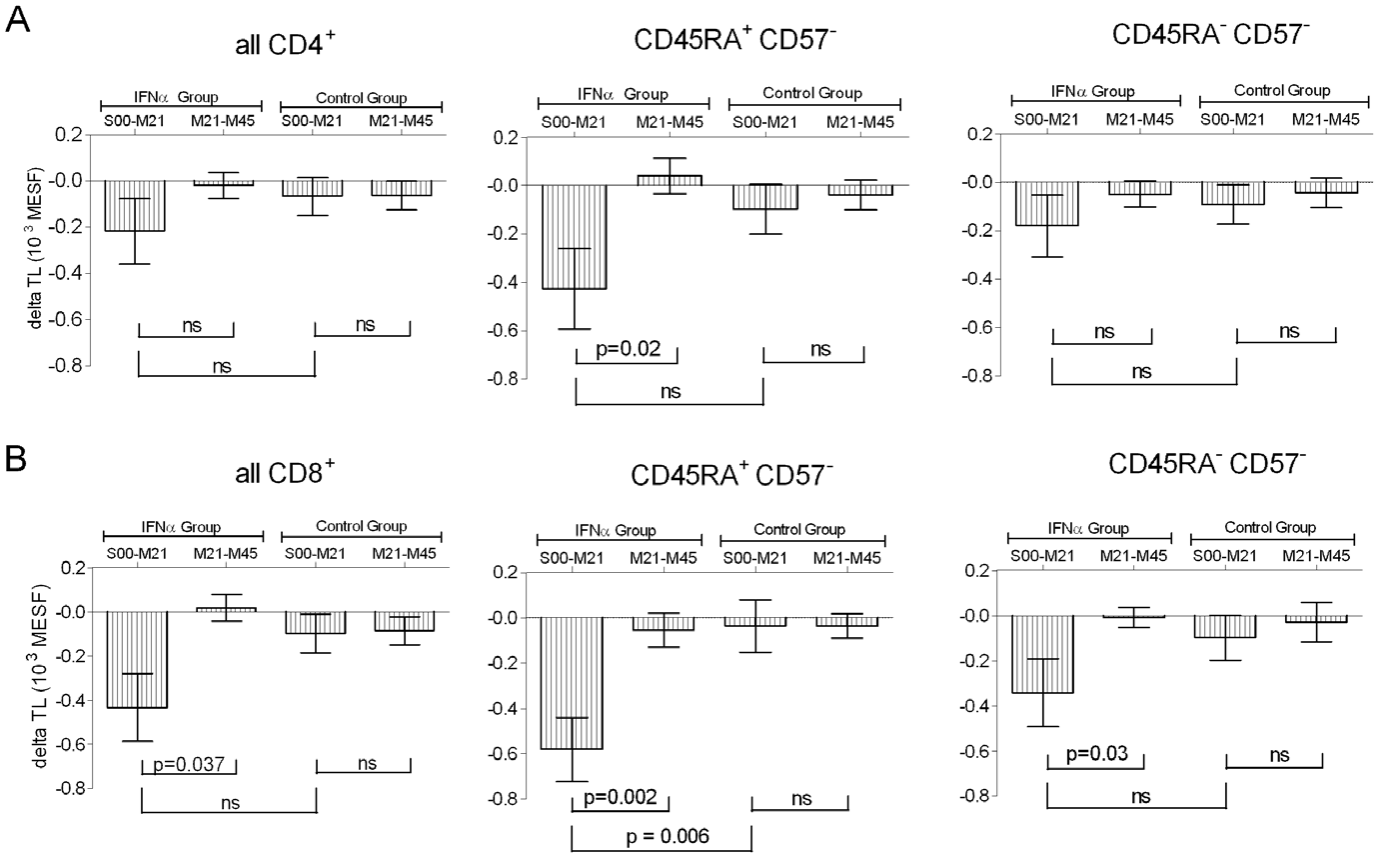
Accelerated telomere length loss (delta TL) occurs in the first 21 months. Delta TL analysis between treatment and control groups for (A) CD4^+^ and (B) CD8^+^ T cells and their naïve and memory subsets for the S00 to M21, and M21 to M45 intervals. P values from Mann-Whitney testing. Error bars are mean ± standard error.

The mid-point (M21) samples allowed assessment of whether the peg-IFNα effect on TL was linear over the four year study period. Within the peg-IFNα group, TL loss was greater in the first interval, S00 to M21, in the naïve CD8^+^ (p = 0.002), memory CD8^+^ (p = 0.03) and naive CD4^+^ subsets (p = 0.02) compared to the M21 to M45 interval (Fig. 4). Naïve CD8^+^ T cell TL loss was higher in the treatment compared to control group in the first interval (p = 0.006), but not different in the second interval. Taken together, these analyses indicate that TL loss accelerated with peg-IFNα treatment, but only in the first interval.

### Age dependence of accelerated telomere length loss

**Figure 5:**
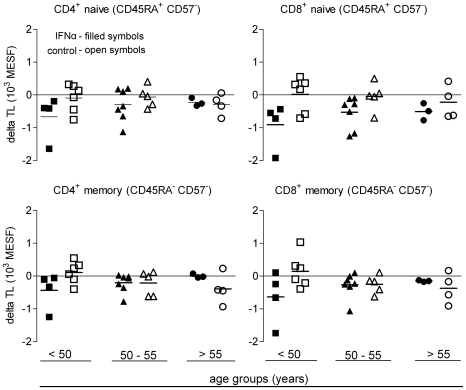
TL loss with therapy was lost with increasing age in a T cell subset-dependent manner. Naïve CD4^+^ (upper left plot), memory CD4^+^ (lower left plot), naïve CD8^+^ (upper right plot), and memory CD8^+^ (lower right plot) T cell change in telomere length (delta TL) from screening to month 45 from subjects in both treatment groups. Each symbol is an individual subject's delta TL for that T cell subset. Filled symbols are IFNα treatment group subjects, open symbols are control group subjects. Horizontal bars are mean values.

To assess the effects of age, treatment group, and TL changes, mixed-effects statistical analyses were performed with results indicating a significant age-related IFN-effect on TLs (data not shown). However, linear model fitting assumptions inherent to a multivariate, mixed effects model were not consistent with the non-linear TL loss data across the three time points. We then partitioned the data set into three age categories: <50 years, 50 to 55 years, and >55 years of age at screening (Fig. 5). The 50–55 year partition was centered on the average age of our subjects (Table 1), with the age partition boundaries selected to roughly balance the numbers of subjects in each partition. This age-partitioned analysis corroborated the mixed-effects model results that accelerated TL loss in the peg-IFNα group was seen in subjects <50 years old and was lost with increasing age in both CD4^+^ and CD8^+^ naïve and memory compartments.

### No difference in telomerase activity

We asked whether effects of long-term IFN therapy on telomerase activity (TA) could explain the accelerated TL loss. As resting peripheral blood T cells directly ex vivo express little or no detectable TA, we analyzed TA after in vitro activation as an indication of a possible durable effect of peg-IFNα therapy (see supporting file Methods S1 for details). No difference in TA as a result of in vitro activation was detected between the two treatment groups (Fig. S2).

### Serum ALT-AST values inversely correlated with TL changes in the control group

**Figure 6:**
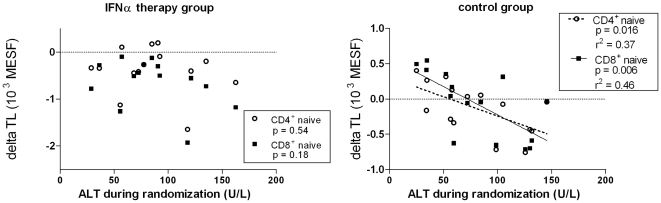
Serum ALT correlates with changes in naïve T cell telomere lengths in the control group. Average serum alanine aminotransferase (ALT) levels during the randomization phase correlated with telomere length loss (delta TL) in the no-therapy control group (right-hand panel), but not in the IFN therapy group (left-hand panel). Delta TL shown is from screening (S00) to month 45 (M45).

Serum alanine aminotransferase (ALT) and serum aspartate aminotransferase (AST) values are commonly used in the clinical setting to monitor on-going liver pathology. Here, we found TL loss (S00 to M45) in naïve CD4^+^ T cells and CD8^+^ T cells negatively correlated with the average serum ALT levels (p = 0.016 and p = 0.006, respectively) during the randomization phase in the control but not the peg-IFNα group (Fig. 6). Average AST levels during randomization also inversely correlated with TL loss in naïve CD4^+^ T cells and CD8^+^ T cells (p = 0.03 and p = 0.006, respectively) during the randomization phase in the control but not the peg-IFNα group (data not shown). These ALT-AST associations with telomere loss were not seen in the memory (CD45RA^−^ CD57^−^) subsets (p >0.1, data not shown).

### Sustained IFNα therapy suppressed T_EMRA_ expansion

**Figure 7:**
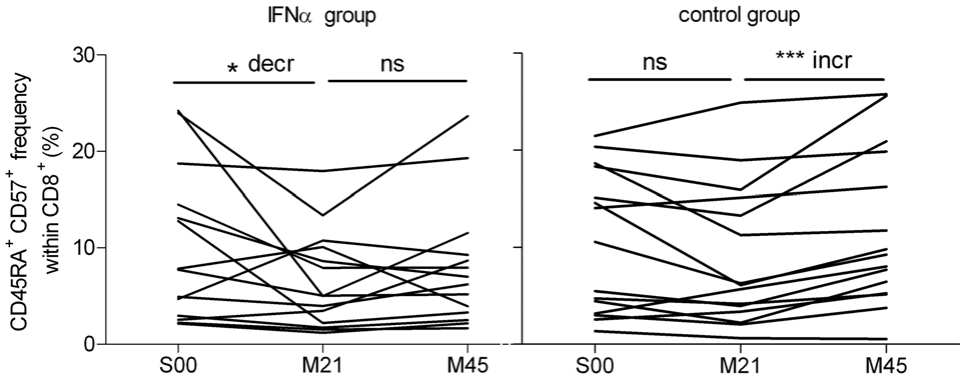
Sustained interferon therapy associated with suppression of CD8^+^ CD45RA^+^ CD57^+^ expansions. CD45RA^+^ CD57^+^ subset frequency (%) within CD8^+^ T lymphocytes across the three time points for the peg-IFNα subjects (left panel) and the no-therapy, control subjects (right panel). Individual lines on the plots represent values from each subject across the three time points. * p<0.05; *** p = 0.0001 by Wilcoxon paired analysis (decr = decrease; incr = increase; ns = not significant).

Analysis of the CD57^+^ T cell subsets indicated these CD4^+^ and CD8^+^ T cell sub-populations did not incur accelerated TL loss in the therapy group (data not shown). This result would be consistent with previously published reports of CD57^+^ as a marker of a T cell with a limited replicative capacity, and thus a limited ability to further erode telomere length [26], [27]. Oligoclonal CD8^+^ CD57^+^ T cell expansions have been reported as a marker for reduced interferon therapy responses in chronic HCV patients [28]. Thus we undertook further analyses of the changes in the frequency of the CD57^+^ subsets within our cHCV cohort. The CD8^+^ CD45RA^+^ CD57^+^ subset, which is primarily a well-described T_EMRA_ population of highly differentiated T cells [27], initially declined in frequency (p = 0.042) in the peg-IFNα group between S00 and M21, followed by no significant change from M21 to M45 (Fig. 7 left panel). In contrast, the control group showed no significant change in the CD8^+^ CD45RA^+^ CD57^+^ T cell frequency between S00 and M21, followed by an increase between M21 and M45 (p = 0.0001; Fig. 7 right panel).

## Discussion

In subjects with cHCV infection, sustained peg-IFNα therapy (90 µg/week) was associated with an increased rate of TL loss in both CD4^+^ and CD8^+^ T cell subsets. Additionally, based on a single intermediate time-point, this IFNα therapy-enhanced TL loss was fully concentrated in the initial 21 months. The delineation of TL changes in T cell CD45RA^+/−^/CD57^−^ subsets shows that declines in the T cell TL are not shifts in naïve (long TL) versus memory (shortened TL) T cell proportions, but true decreases in TL with the greatest impact on the naïve T cell subsets. The lack of an accelerated TL loss effect in naïve T cells in the second interval in the peg-IFNα group suggests that a new homeostasis is reached. Our findings are consistent with the telomere erosion effects of increased lymphocyte turn-over in response to a sustained lymphopenic signal as predicted from the mathematical models of de Boer and Noest [29]. Increased T cell turn-over would also account for our observation that sustained peg-IFNα treatment inhibited the expansion of CD8^+^ CD45RA^+^ CD57^+^ T_EMRA_ cells, a phenomenon observed in the control group.

By HALT-C trial design, subjects in both groups received combination therapy with peg-IFNα (180 µg/week) plus ribavirin for the initial 24 weeks of the study. PBMC were not available from other intermediate time points. Therefore, it is possible that accelerated TL loss occurred in both groups during the initial phase of lead-in therapy. Average TL could then have rebounded in the control group between the end of therapy at week 24 and month 21 whereas ongoing, lower-dose peg-IFNα therapy maintained the lower average TL (or suppressed a TL recovery) in the therapy group. Consistent with this interpretation, the decline in TL was slower during the second interval than in the earlier interval in the therapy group. If this interpretation is correct, it is impossible to determine whether the effect of the initial therapy on TL is attributable to the higher dose of peg-IFNα or to ribavirin. Nevertheless, sustained peg-IFNα was clearly associated with accelerated TL loss during the subsequent 3½ years in comparison with the control group.

An important finding from our study is the correlation of baseline T cell TL in these cHCV subjects with viremia and BMI. It should be noted that subjects in the randomization phase of the trial had endured HCV viremia for decades (range: 14–51 years) and failed to achieve SVR during combination peg-IFNα-ribavirin therapy. The absence of a negative correlation of baseline T cell TL with age in this cohort may therefore reflect stronger impacts of long-term viremia and the generally high levels of obesity in these subjects.

We speculate that the negative association between serum viral RNA levels and baseline TL reflects chronic elevated T cell activation, cell death, and proliferation due to persistent presentation of HCV antigens in a dose-dependent manner. Alternatively, higher circulating viral RNA levels may also drive greater endogenous type I IFN production. A negative correlation of BMI with baseline TL in naïve T cells could also reflect chronic inflammation in obesity [30]. Chronic inflammation as a result of obesity may lead to decreased thymic output [31], inducing increased homeostatic proliferation of naïve T lymphocytes and thus a decrease in TL through replicative erosion.

Another possible explanation for the lack of an age-dependent TL association in this cohort relates to evidence that short telomeres play a causal role in a variety of age-related diseases [32], [33], [34], [35]. Thus it is plausible that study inclusion criteria biased the enrolled study population away from subjects with very short telomeres.

Elevated ALT levels in cHCV are indicative of hepatocellular inflammation and necrosis [12]. Our finding of a correlation of serum ALT levels with naïve T cell TL loss in the control group likely reflects T cell responses to infected HCV-infected hepatocytes and the extended time frame over which telomere lengths were analyzed. This relationship was possibly obscured in the peg-IFNα group by the effects of continuous therapy. The correlation between TL changes and ALT, which could be due to T cell clonal exhaustion, immunosenescence, or a combination of these and/or other immunological factors, suggests that normalization of liver enzymes levels in the blood may coincide with a reduction in T cell turn-over and thus reduced telomere erosion.

We hypothesized that maintenance peg-IFNα therapy would cause increased TL loss in T cells as a result of inhibition of telomerase activity [4], [36]. However, we did not find a significant difference between the peg-IFNα and control groups in telomerase activity in PBMC stimulated in vitro. It is quite possible that using in vitro-stimulated PBMC the telomerase assay fails to detect an inhibitory effect of peg-IFNα therapy that existed in vivo. In any case, a telomerase inhibition mechanism falls short in explaining the predominance of an accelerated TL loss on naïve T cells in these older adults where thymic output of new, longer telomere naïve T cells is thought to be neglible.

We further found a decline in the peg-IFNα effect on TL loss with increasing age. Increasing age is associated with increased failure rates of IFN therapy for cHCV [14]. This may suggest that elevated TL erosion caused by peg-IFNα is counteracted by replicative senescence deferentially within T cell compartments. The delineation of TL loss effect by CD4^+^ and CD8^+^ T cell subset by age group results suggest a hierarchy of age-dependent T cell senescence in chronic HCV patients. Thus senescence may occur in the order: memory CD4^+^>memory CD8^+^>naïve CD4^+^>naïve CD8^+^ in this cHCV setting. Further, this result of the memory CD4^+^ T cell compartment becoming refractory to IFN-induced TL decreases as a marker of immunosenescence onset is consistent with the finding by Hoare, et al. where they found TL in CD4^+^ CD45RO^+^ memory T cells was a stronger predictor of SVR with IFN therapy than TL in any other T cell subset [37].

Enhanced T cell TL loss in subjects who received long-term peg-IFNα therapy suggests that T cells in these subjects have reduced proliferative reserve. Subjects receiving type I IFN therapy are known to be more susceptible to bacterial infections; this has been attributed to neutropenia, but several studies have shown no temporal correlation between neutrophil count and infections [38], [39]. Diminished memory T cells and proliferative reserve related to naïve T cell TL loss, as shown in this study, could contribute to the increased susceptibility to infection and disease while on interferon therapy. Importantly, diminished naive T cell proliferative TL reserve incurred under sustained IFN therapy may persist well beyond the end of therapy. Indeed, as age-related thymic involution severely limits production of new, long telomere, naïve T cells [37], a sustained accelerated TL erosion thus may leave a permanently degraded naïve T cell compartment. Support for this possibility comes from a recent analysis of a subset of patients from the HALT-C cohort prospectively followed for more than 5 years after the trial. That study showed rates of non-liver-related death were significantly higher (p = 0.01) among patients with liver fibrosis who received the 3½ year peg-IFNα therapy compared to similar patients in the control arm [40].

Although extended type I interferon therapy beyond 48 weeks in the cHCV settings is not typically warranted, there are additional clinical settings where extended interferon therapy is utilized. Some examples include relapsing-remitting multiple sclerosis and melanoma [41], [42]. Accordingly, as extended type I interferon is clinically practiced, our findings suggest that additional studies of the effects of peg-IFNα therapy on age-related T cell senescence are warranted.

## Materials and Methods

### Ethics Statement

All subjects provided written, informed consent for participation under protocols approved by the institutional review boards of all participating study centers and conformed to the ethical guidelines of the 1975 Declaration of Helsinki. Specifically, the human-derived PBMC samples used in this telomere length HALT-C ancilliary study came from the following institutions with formal Institutional Review Board approval obtained from all: Human Subjects/IRB, University of Massachusetts Medical School, Worcester, MA; Human Subjects Protection Office, University of Connecticut Health Center, Farmington, CT; Biomedical IRB, Saint Louis University, Saint Louis, MO; Institutional Review Board, The University of Texas Southwestern Medical Center, Dallas, TX; Institutional Review Board, University of Southern California Health Sciences Campus, Los Angeles, CA; Institutional Review Board for Human Subject Research, University of Michigan Medical School, Ann Arbor, MI.

### Subjects and study design

The HALT-C trial design has been described in detail elsewhere [10]. Initial enrollment criteria required all subjects to have histologically-confirmed liver fibrosis or cirrhosis (Ishak score ≥3). Subjects who remained viremic after 6 months of peg-IFNα plus ribavirin therapy were randomized either to continued maintenance-dose (90 µg/week) peg-IFNα for an additional 3.5 years or a monitor-only control group, for a total study duration of 48 months per patient. Neither subjects nor clinicians were blinded to treatment assignment. Peripheral blood mononuclear cell (PBMC) samples for telomere length analysis came from a representative subset of the HALT-C cohort consisting of 29 patients who successfully completed the 48 month randomization phase from three time points within the study period: screening (S00), month 21 (M21), and month 45 (M45).

### PBMC samples

Subjects from each randomization group, peg-IFNα treatment and control, were selected as matched-pairs, based on age, gender, and Ishak fibrosis score (Table 1). Subjects for the peg-IFNα therapy group were selected for high compliance (>80%). For TL analysis, we were blinded to treatment group assignment, patient characteristics, and chronological order for each patient's PBMC until after the completed TL data set was returned to the HALT-C Data Coordinating Center. Patient data (Table 1) included: age at enrollment and PBMC collections, gender, race, body mass index (BMI) at enrollment, estimated duration of HCV infection, serum alanine amino transferase (ALT) levels, serum HCV RNA levels, and Ishak fibrosis score [15], [43], [44]. Additionally, PBMC from a separate, healthy cohort of 19 subjects matching the age range of the HALT-C subjects were collected with written, informed consent under University of Massachusetts Medical School Institutional Review Board-approved protocols.

### Telomere length measurements

TL was measured using a modified flowFISH assay [21]. 4×10^6^ PBMC from each sample were stained with Alexa700-anti-hCD4 and APC-Alexa750-anti-hCD8 (eBiosciences, San Diego, CA), treated with 1 mM suberic acid bis(3-sulfo-N-hydroxysuccinimide ester) sodium salt crosslinker then quenched with 50 mM Tris-HCl. Cells were fixed in 4% formaldehyde and 0.05% saponin for 25 minutes at 4°C. All samples were incubated in lithium-based RNase buffer plus 0.05% saponin with 2 µL RNase1 (Promega, Madison, WI) for two hours. Samples were then divided to three probe (+) tubes and one probe (−) tube for hybridization in 70% formamide, 150 mM lithium-chloride buffer at 82°C for 12 minutes. Probe (+) tubes contained Cy5-OO-(CCCTAA)_3_-EE peptide nucleic acid probe at 0.5 µg/mL. After overnight cooling, samples were washed twice in 70% formamide buffer then with 2 mL PBS, stained with PE-Cy7-anti-hCD45RA antibody and PE-anti-hCD57, and resuspended in PBS-BSA containing 0.1 µg/mL 4′,6-diamidino-2-phenylindole, dihydrochloride (DAPI).

### Sample analysis

Samples were analyzed with a FACS-Aria (BD, San Jose, CA) cytometer with performance verified prior to each assay using calibration beads. A minimum of 20,000 lymphocyte-gated events were collected for every tube. Each sample probe (−) tube mean fluorescence intensity (MFI) was subtracted from the average MFI of the matching three probe (+) tubes to obtain a specific MFI. A minimum of 30 events per gated population was employed to include a resulting MFI in an analysis; this limit only affected CD57^+^ subsets. Specific MFI values were converted to molecules of equivalent soluble fluorescence (MESF) using linear calibration beads-derived MFI to MESF linear equation. A healthy donor PBMC sample analyzed in triplicate in four of the eight total flow FISH analyses provided inter-assay and intra-assay coefficients of variation (CV). Inter-assay CV for all CD4+ and all CD8+ T cells were 7.4% and 6.6% respectively. Intra-assay CV for all CD4^+^ and all CD8^+^ T cells were 1.1% and 1.3% respectively. PBMC samples from each subject's three time points were run in the same assay to minimize inter-assay variation.

### Telomerase activity measurement in *in vitro* activated T lymphocytes

See supporting file Methods S1.

### Data and statistical analysis

Although subjects within the two randomization groups were initially selected as matched pairs, TL data from one subject was unusable and therefore unpaired analyses were conducted. For linear regression analyses, fitted lines, correlation values, and p values are from linear regression testing. Statistical analyses were performed using Prism v5.0 (Graphpad, LaJolla, CA) with p values<0.05 considered statically significant. All statistical tests were two-tailed.

## Supporting Information

Figure S1 **Baseline telomere lengths and CD57^+^ frequencies were not different at screening between the two groups.** (A) Subject telomere lengths at screening. Each symbol is an individual subject's TL measured by flowFISH in that T cell subset. P values are from unpaired *t* test analysis. Horizontal bars are mean values. (B) CD57^+^ subset distribution within respective CD4^+^ and CD8^+^ T cell populations from screening (S00), month 21 (M21), and month 45 (M45). Plots are box and whiskers 5–95 percentile bar graphs showing outlier values; peg-IFNα therapy subjects shown as empty bars; control group subjects, filled bars. RA^+^ or RA^−^ indicates CD45RA^+^ or CD45RA^−^ respectively, 57^+^ indicates CD57^+^.
(JPG)

Figure S2 **Induced telomerase activity in PBMC between treatment groups was not different at any time point.** Telomerase activity (TA) was assessed in *in vitro* stimulated PBMC from each of the three time points, screening (S00), month 21 (M21), and month 45 (M45), and the results analyzed between treatment groups as shown. Statistical p values are from Mann-Whitney non-parametric analysis. PBMC were stimulated with plate-bound anti-CD3 plus anti-CD28 for 3 days and then tested for TA by a commercial real-time PCR-based TRAP assay as described in Methods S1.
(JPG)

Methods S1 **Telomerase activity measurement in in vitro activated T lymphocytes.**
(DOC)

## References

[1] de Lange T (2005). Shelterin: the protein complex that shapes and safeguards human telomeres.. Genes Dev.

[2] Hodes RJ, Hathcock KS, Weng NP (2002). Telomeres in T and B cells.. Nat Rev Immunol.

[3] Weng NP (2008). Telomere and adaptive immunity.. Mech Ageing Dev.

[4] Xu D, Erickson S, Szeps M, Gruber A, Sangfelt O (2000). Interferon alpha down-regulates telomerase reverse transcriptase and telomerase activity in human malignant and nonmalignant hematopoietic cells.. Blood.

[5] Hirsch RL, Johnson KP (1986). The effects of long-term administration of recombinant alpha-2 interferon on lymphocyte subsets, proliferation, and suppressor cell function in multiple sclerosis.. J Interferon Res.

[6] Boyman O, Letourneau S, Krieg C, Sprent J (2009). Homeostatic proliferation and survival of naive and memory T cells.. Eur J Immunol.

[7] Hoofnagle JH, Seeff LB (2006). Peginterferon and ribavirin for chronic hepatitis C.. N Engl J Med.

[8] Lok AS, Seeff LB, Morgan TR, di Bisceglie AM, Sterling RK (2009). Incidence of hepatocellular carcinoma and associated risk factors in hepatitis C-related advanced liver disease.. Gastroenterology.

[9] Di Bisceglie AM, Shiffman ML, Everson GT, Lindsay KL, Everhart JE (2008). Prolonged therapy of advanced chronic hepatitis C with low-dose peginterferon.. N Engl J Med.

[10] Lee WM, Dienstag JL, Lindsay KL, Lok AS, Bonkovsky HL (2004). Evolution of the HALT-C Trial: pegylated interferon as maintenance therapy for chronic hepatitis C in previous interferon nonresponders.. Control Clin Trials.

[11] Everhart JE, Lok AS, Kim HY, Morgan TR, Lindsay KL (2009). Weight-related effects on disease progression in the hepatitis C antiviral long-term treatment against cirrhosis trial.. Gastroenterology.

[12] Jamal MM, Soni A, Quinn PG, Wheeler DE, Arora S (1999). Clinical features of hepatitis C-infected patients with persistently normal alanine transaminase levels in the Southwestern United States.. Hepatology.

[13] Lin R, Liddle C, Byth K, Farrell GC (1996). Virus and host factors are both important determinants of response to interferon treatment among patients with chronic hepatitis C.. J Viral Hepat.

[14] Romeo R, Rumi M, Colombo M (1995). Alpha interferon treatment of chronic hepatitis C.. Biomed Pharmacother.

[15] Rothman AL, Morishima C, Bonkovsky HL, Polyak SJ, Ray R (2005). Associations among clinical, immunological, and viral quasispecies measurements in advanced chronic hepatitis C.. Hepatology.

[16] Vandelli C, Renzo F, Braun HB, Tisminetzky S, Albrecht M (1999). Prediction of successful outcome in a randomised controlled trial of the long-term efficacy of interferon alpha treatment for chronic hepatitis C.. J Med Virol.

[17] Tillmann HL, Manns MP, Rudolph KL (2005). Merging models of hepatitis C virus pathogenesis.. Semin Liver Dis.

[18] Valdes AM, Andrew T, Gardner JP, Kimura M, Oelsner E (2005). Obesity, cigarette smoking, and telomere length in women.. Lancet.

[19] Zannolli R, Mohn A, Buoni S, Pietrobelli A, Messina M (2008). Telomere length and obesity.. Acta Paediatr.

[20] O'Callaghan NJ, Clifton PM, Noakes M, Fenech M (2009). Weight loss in obese men is associated with increased telomere length and decreased abasic sites in rectal mucosa.. Rejuvenation Res.

[21] Baerlocher GM, Vulto I, de Jong G, Lansdorp PM (2006). Flow cytometry and FISH to measure the average length of telomeres (flow FISH).. Nat Protoc.

[22] Azzalin CM, Reichenbach P, Khoriauli L, Giulotto E, Lingner J (2007). Telomeric repeat containing RNA and RNA surveillance factors at mammalian chromosome ends.. Science.

[23] Koch S, Larbi A, Derhovanessian E, Ozcelik D, Naumova E (2008). Multiparameter flow cytometric analysis of CD4 and CD8 T cell subsets in young and old people.. Immun Ageing.

[24] Weng NP, Levine BL, June CH, Hodes RJ (1995). Human naive and memory T lymphocytes differ in telomeric length and replicative potential.. Proc Natl Acad Sci U S A.

[25] Aubert G, Lansdorp PM (2008). Telomeres and aging.. Physiol Rev.

[26] Brenchley JM, Karandikar NJ, Betts MR, Ambrozak DR, Hill BJ (2003). Expression of CD57 defines replicative senescence and antigen-induced apoptotic death of CD8+ T cells.. Blood.

[27] Le Priol Y, Puthier D, Lecureuil C, Combadiere C, Debre P (2006). High cytotoxic and specific migratory potencies of senescent CD8+ CD57+ cells in HIV-infected and uninfected individuals.. J Immunol.

[28] Manfras BJ, Weidenbach H, Beckh KH, Kern P, Moller P (2004). Oligoclonal CD8+ T-cell expansion in patients with chronic hepatitis C is associated with liver pathology and poor response to interferon-alpha therapy.. J Clin Immunol.

[29] De Boer RJ, Noest AJ (1998). T cell renewal rates, telomerase, and telomere length shortening.. J Immunol.

[30] Hotamisligil GS, Erbay E (2008). Nutrient sensing and inflammation in metabolic diseases.. Nat Rev Immunol.

[31] Dixit VD (2008). Adipose-immune interactions during obesity and caloric restriction: reciprocal mechanisms regulating immunity and health span.. J Leukoc Biol.

[32] Gilley D, Herbert BS, Huda N, Tanaka H, Reed T (2008). Factors impacting human telomere homeostasis and age-related disease.. Mech Ageing Dev.

[33] Farzaneh-Far R, Lin J, Epel E, Lapham K, Blackburn E (2009). Telomere length trajectory and its determinants in persons with coronary artery disease: longitudinal findings from the heart and soul study.. PLoS One.

[34] Huzen J, de Boer RA, van Veldhuisen DJ, van Gilst WH, van der Harst P (2010). The emerging role of telomere biology in cardiovascular disease.. Front Biosci.

[35] Zee RY, Michaud SE, Germer S, Ridker PM (2009). Association of shorter mean telomere length with risk of incident myocardial infarction: a prospective, nested case-control approach.. Clin Chim Acta.

[36] Reed JR, Vukmanovic-Stejic M, Fletcher JM, Soares MV, Cook JE (2004). Telomere erosion in memory T cells induced by telomerase inhibition at the site of antigenic challenge in vivo.. J Exp Med.

[37] Hoare M, Gelson WT, Das A, Fletcher JM, Davies SE (2010). CD4+ T-lymphocyte telomere length is related to fibrosis stage, clinical outcome and treatment response in chronic hepatitis C virus infection.. J Hepatol.

[38] Soza A, Everhart JE, Ghany MG, Doo E, Heller T (2002). Neutropenia during combination therapy of interferon alfa and ribavirin for chronic hepatitis C.. Hepatology.

[39] Antonini MG, Babudieri S, Maida I, Baiguera C, Zanini B (2008). Incidence of neutropenia and infections during combination treatment of chronic hepatitis C with pegylated interferon alfa-2a or alfa-2b plus ribavirin.. Infection.

[40] Di Bisceglie AM, Stoddard AM, Dienstag JL, Shiffman ML, Seeff LB Excess mortality in patients with advanced chronic hepatitis C treated with long-term peginterferon.. Hepatology.

[41] Hauschild A, Gogas H, Tarhini A, Middleton MR, Testori A (2008). Practical guidelines for the management of interferon-alpha-2b side effects in patients receiving adjuvant treatment for melanoma: expert opinion.. Cancer.

[42] Lam S, Wang S, Gottesman M (2008). Interferon-beta1b for the treatment of multiple sclerosis.. Expert Opin Drug Metab Toxicol.

[43] Delgado-Borrego A, Jordan SH, Negre B, Healey D, Lin W (2010). Reduction of insulin resistance with effective clearance of hepatitis C infection: results from the HALT-C trial.. Clin Gastroenterol Hepatol.

[44] Everhart JE, Wright EC, Goodman ZD, Dienstag JL, Hoefs JC (2010). Prognostic value of Ishak fibrosis stage: Findings from the hepatitis C antiviral long-term treatment against cirrhosis trial.. Hepatology.

