# Supplementary figures and images for: Extended Interferon-Alpha Therapy Accelerates Telomere Length Loss in Human Peripheral Blood T Lymphocytes
Source: PLoS One. 2011 Aug 4;6(8):e20922. doi: 10.1371/journal.pone.0020922 (PMC3150344; doi:10.1371/journal.pone.0020922)

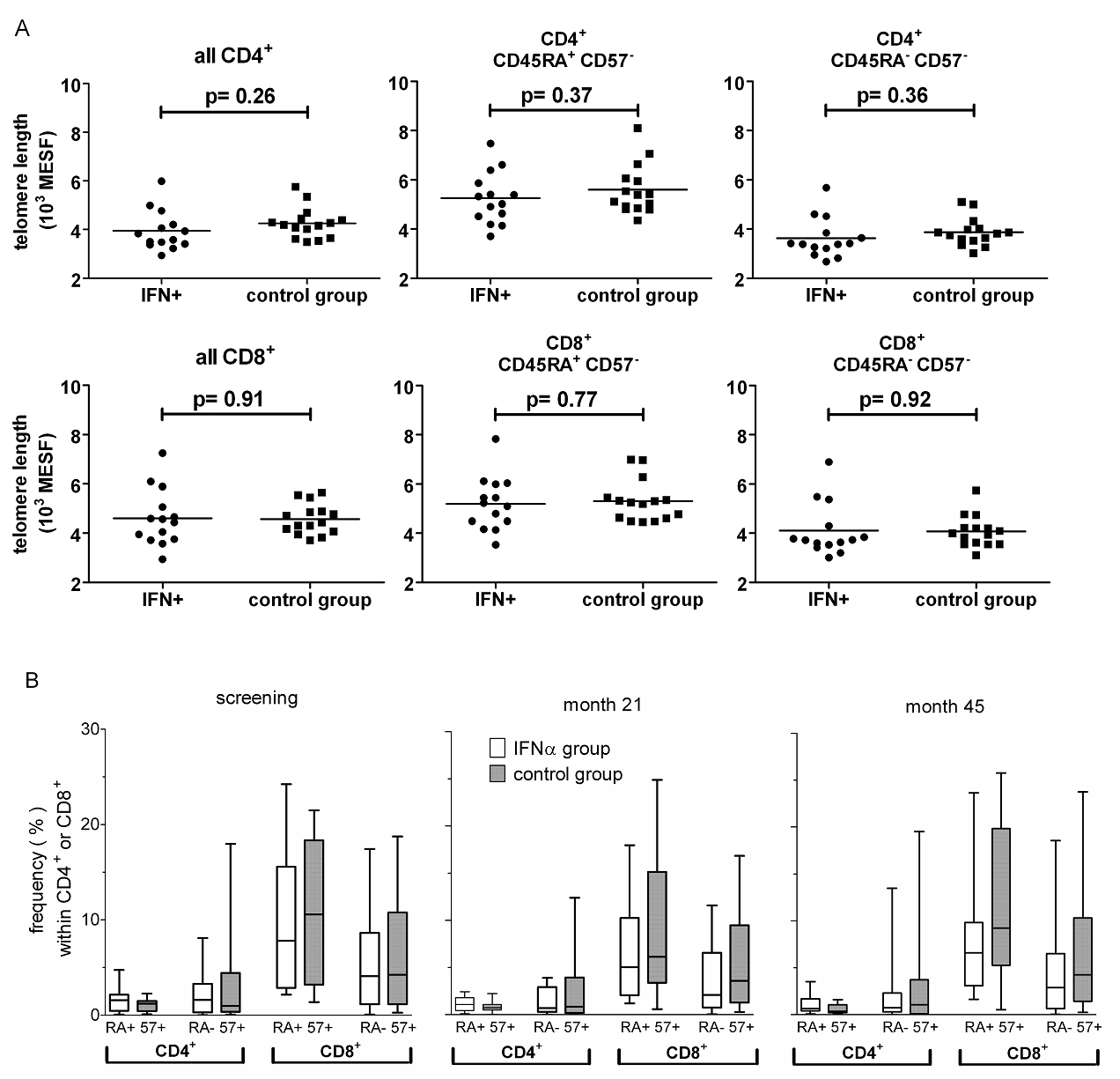

Supplement: Figure S1 — Baseline telomere lengths and CD57+ frequencies were not different at screening between the two groups. (A) Subject telomere lengths at screening. Each symbol is an individual subject's TL measured by flowFISH in that T cell subset. P values are from unpaired t test analysis. Horizontal bars are mean values. (B) CD57+ subset distribution within respective CD4+ and CD8+ T cell populations from screening (S00), month 21 (M21), and month 45 (M45). Plots are box and whiskers 5–95 percentile bar graphs showing outlier values; peg-IFNα therapy subjects shown as empty bars; control group subjects, filled bars. RA+ or RA− indicates CD45RA+ or CD45RA− respectively, 57+ indicates CD57+. (JPG) [file pone.0020922.s001.jpg]

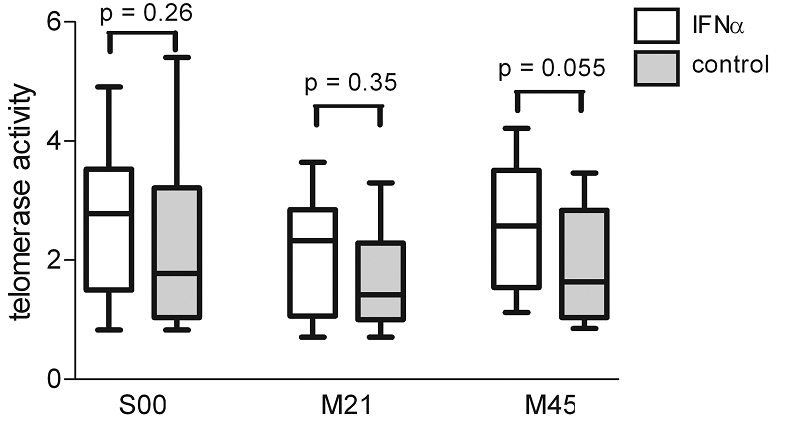

Supplement: Figure S2 — Induced telomerase activity in PBMC between treatment groups was not different at any time point. Telomerase activity (TA) was assessed in in vitro stimulated PBMC from each of the three time points, screening (S00), month 21 (M21), and month 45 (M45), and the results analyzed between treatment groups as shown. Statistical p values are from Mann-Whitney non-parametric analysis. PBMC were stimulated with plate-bound anti-CD3 plus anti-CD28 for 3 days and then tested for TA by a commercial real-time PCR-based TRAP assay as described in Methods S1. (JPG) [file pone.0020922.s002.jpg]
