# Supplementary material for: Extended Interferon-Alpha Therapy Accelerates Telomere Length Loss in Human Peripheral Blood T Lymphocytes
Source: PLoS One. 2011 Aug 4;6(8):e20922. doi: 10.1371/journal.pone.0020922 (PMC3150344; doi:10.1371/journal.pone.0020922)
Supplement: Methods S1 — Telomerase activity measurement in in vitro activated T lymphocytes. (DOC) [file pone.0020922.s003.doc]

# Supplemental methods S1 for Figure S2:

# Extended interferon-alpha therapy accelerates telomere loss in human peripheral blood T lymphocytes

Joel M. O'Bryan,^1^ James A. Potts,^1^ Herbert L. Bonkovsky,^2,3^ Anuja Mathew,^1*^ and Alan L. Rothman^1,4^ for the HALT-C Trial Group^†^

^1^Department of Medicine, University of Massachusetts Medical School, Worcester, Massachusetts, USA

^2^Department of Medicine, University of Connecticut Health Center, Farmington, Connecticut, USA

^3^Carolinas Medical Center, Charlotte, North Carolina, USA 28203

^4^Institute for Immunology and Informatics, University of Rhode Island, Providence, Rhode Island, USA

^†^ This is publication number 61 from the HALT-C Trial Study Group.

* Corresponding author:

Anuja Mathew Ph.D.

University of Massachusetts Medical School

Room S6-862

55 Lake Avenue North

Worcester, MA 01655

Email: Anuja.mathew@umassmed.edu

Phone: 508-856-4182

FAX: 508-856-4890

**Registrations**

The HALT-C trial was registered with clinicaltrials.gov (#NCT00006164).

**In vitro PBMC activation and telomerase activity measurement.**

Telomerase activity in stimulated PBMC was measured using a commercial PCR-based, real-time telomerase repeat activity protocol kit (RT-TRAP, Millipore, Billerica, MA) per manufacturer’s instructions. Briefly, 10^6^ PBMC from each patient time point sample were stimulated for three days with plate-bound anti-CD3 (clone OKT3, BD) and anti-CD28 (clone 28.1, BD) each at 3 μg/mL in 2 mL complete RPMI-1640 media (Gibco-Invitrogen) with 10% fetal calf serum. Cell lysate extracts were prepared from harvested cells pellets and placed in -80^o^C frozen aliquots per manufacturer’s instructions using provided CHAPS lysis buffer. RT-TRAP was performed per manufacturer’s instructions using 2000 cell equivalents per test well on a 96-well PCR plate. Real-time PCR was performed on an ABI Prism 7300 (Applied Biosciences). Using the unit equipped, real-time software (SDS v1.4), triplicate-averaged sample fluorescence threshold crossing (Ct) values were converted, using kit-provided controls and template standards and the resulting standard curve template control values, to a mean telomerase product quantity for each sample. Telomerase activity (TA) results are a ratio of a triplicate-derived mean quantity to the same-plate, negative control mean quantity.
